# Supplementary material for: A Standardized Guide to Developing an Online Grocery Store for Testing Nutrition-Related Policies and Interventions in an Online Setting
Source: Int J Environ Res Public Health. 2021 Apr 24;18(9):4527. doi: 10.3390/ijerph18094527 (PMC8123213; doi:10.3390/ijerph18094527)
Supplement: Supplementary file 1 [file ijerph-18-04527-s001.zip › ijerph-1107833-supplementary.pdf]

FIGURE S1. Shopping list and shopping basket

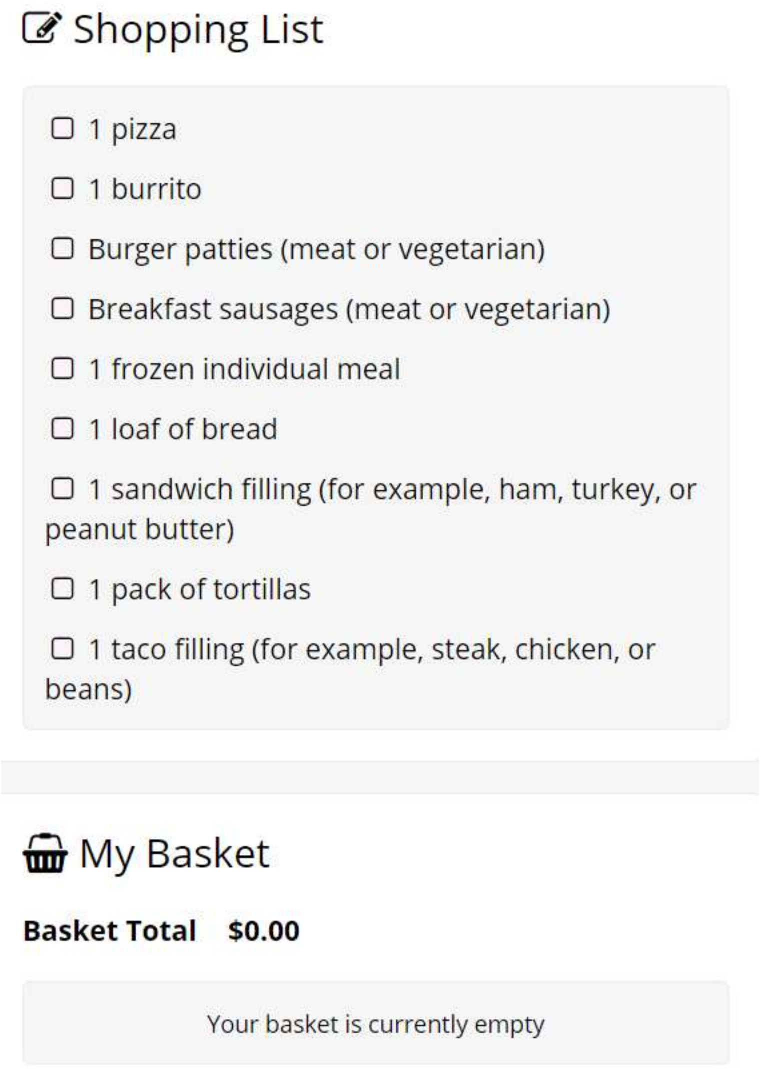

TABLE S1. Data scraping request

**Variable(s)**	**Cleaning request**
Energy (Kcal), Fat, Saturated Fat, Carbohydrates, Sugar, Salt, Fiber, Protein	• Include the tenths place<br>• Put a space between the number and the measurement (e.g., "10.0 g" instead of "10.0g")<br>• When the measurement is grams change grm to g<br>• Change Kcal and Cal to cal<br>• Ensure all numbers have a measurement<br>• Fat, saturated fat, carbs, sugar, fiber, protein should include "g"<br>• Sodium should include "mg"<br>• Calories should include "cal"
Ingredients	• Remove the "ingredients:" from the products that currently contain that text in their ingredient list<br>• Make all ingredients all caps
Product	• Capitalize the first word in all products. (For example, "STOUFFER'S CLASSICS Macaroni & Cheese" would become "Stouffer's Classics Macaroni & Cheese")<br>• For products that begin with "Great Value" or "Freshness Guaranteed" remove these phrases and keep the rest of the product name
Price	• Include the hundredths place for all prices

TABLE S2. Procedures for tagging meat products

We included separate tags for beef, pork, other red meat, and all red meat. To narrow the list of potential red meat products for tagging, we first excluded shelves that would not contain red meat (e.g., Fruit Drinks, Dried Beans); and all products on those shelves were automatically tagged with a "0." For the remaining products, we systematically reviewed the ingredient list to ascertain whether any beef, pork, or other red meat ingredient was present, and assigned such products a "1." For example, sliced ham would be tagged "1" for pork, "0" for beef, and "0" for other red meat. We then tagged products as "red meat" if the product had a tag of "1" for beef, pork, or other red meat. In addition to red meat products, we also tagged processed red meat products and processed non-red meat products. We used both the NOVA and International Agency for Research on Cancer (IARC) definitions of processed foods to determine if a product did or did not contain processed meat.[1,2] We tagged a product as processed red meat if it contained red meat and at least one ingredient used in processes to transform meat. We also tagged a product as processed non-red meat if it contained poultry or seafood and at least one ingredient used in processes to transform meat. We excluded uncooked meats from the processed meat category if they only contained natural flavoring as an additional ingredient (e.g., uncooked pork chops containing only "pork and natural flavorings").(28) See table below for keywords we included or excluded in our search for items to tag.

**Product**	**Keyword**
Red meat	*Include*: Beef, pork, bison, boar, buffalo, elk, goat, lamb, venison, veal

	Exclude: Beef base, beef bone stock, beef bone extract, beef bouillon, beef broth, beef broth concentrate, beef broth flavor, beef broth powder, beef concentrate, beef consommé, beef extract, beef fat, beef flavor, beef flavored, beef flavoring, beef flavors, beef flavour, beef flavouring, beef flavours, beef gelatin, beef gravy, beef juice, beef juices, beef powder, beef seasoning, beef seasonings, beef shortening, beef stock, beef tallow, powdered beef, pork base, pork bouillon, pork broth, pork broth concentrate, pork broth flavor, pork broth powder, pork concentrate, pork consommé, pork extract, pork fat, pork flavor, pork flavored, pork flavoring, pork flavors, pork flavour, pork flavouring, pork flavours, pork gelatin, pork gravy, pork juice, pork juices, pork lard, pork powder, pork seasoning, pork seasonings, pork shortening, pork stock, powdered pork<br><br>NOTE: Some products will list "Beef:" or “Pork:” at the start of the ingredient list, but not contain any actual meat (e.g. ramen). Keep an eye out for this.
Processed red meat	*Include*:<br>Contains one of the following: bacon, sausage, pepperoni, salami, ham, prosciutto, frank, pastrami, roast beef, brat, bologna<br><br>Contains red meat and at least one of the following ingredients: flavoring, flavouring, artificial flavor(ings), artificial flavour(ings), natural flavor(ings), natural flavour(ings), sodium phosphate, potassium lactate, potassium acetate, sodium diacetate, sodium erythorbate, sodium benzoate, sodium propionate, potassium chloride, oleoresin of paprika, sodium nitrite, sodium nitrate, smoke flavoring,

	dextrose, lactic acid starter culture, bha (butylated hydroxyanisole), bht (butylated hydroxytoluene), citric acid, monosodium glutamate (msg), corn syrup, isolated soy product, isolated soy protein, celery powder, celery juice, celery salt, salt, sodium chloride, mechanically separated chicken, mechanically separated pork, mechanically separated turkey<br><br>NOTE: 1) Keep an eye out for vegetarian and poultry/seafood alternatives that contain one of these words. They should not be flagged. 2) If there is an animal meat that is raw and the only other ingredient it contains is “natural flavoring” do not count it as a processed meat.
Processed non-red meat	*Include*: Contains non- red meat (e.g., poultries (chicken, turkey, hen, guinea fowl, duck) and seafood (fish, shrimp, scallop, crab, lobster, mussel, clam, oyster, squid, and octopus)) and at least one of the following ingredients: flavoring, flavouring, artificial flavor(ings), artificial flavour(ings), natural flavor(ings), natural flavour(ings), sodium phosphate, potassium lactate, potassium acetate, sodium diacetate, sodium erythorbate, sodium benzoate, sodium propionate, potassium chloride, oleoresin of paprika, sodium nitrite, sodium nitrate, smoke flavoring, dextrose, lactic acid starter culture, bha (butylated hydroxyanisole), bht (butylated hydroxytoluene), citric acid, monosodium glutamate (msg), corn syrup, isolated soy product, isolated soy protein, celery powder, celery juice, celery salt, salt, sodium chloride, mechanically separated chicken, mechanically separated pork, mechanically separated turkey

	NOTE: If there is an animal meat that is raw and the only other ingredient it contains is “natural flavoring” do NOT count it as a processed meat.

[1]Monteiro, C.A., Cannon, G., Lawrence, M., Costa Louzada, M.L. and Pereira Machado, P. 2019. Ultra-processed foods, diet quality, and health using the NOVA classification system. Rome, FAO.

[2]Bouvard V, Loomis D, Guyton KZ, Grosse Y, El Ghissassi F, Benbrahim-Tallaa L, et al. Carcinogenicity of consumption of red and processed meat. The Lancer Oncology. 2015; 16(16):1599-1600.

TABLE S3. Procedures for tagging eligible fruit and vegetables

We tagged fruit and vegetable products eligible for a 50% discount or being added as a default shopping cart item using the inclusion and exclusion criteria listed in the table below. We tagged the following products with a "1": fresh and frozen fruits and vegetables without added sugars, fats, oils, or salt; and canned fruits and vegetables with no added sugars, fats, or oils, and ≤141 milligrams of sodium per serving (i.e., low-sodium per Food and Drug Administration guidelines).(29) We tagged all other fruit and vegetable products as "0," including dried fruits and vegetables; fruit and vegetable beverages, including juices and "squeezables"; fruit leathers; prepared produce intended for off-premises consumption (e.g., pre-sliced mango); and products containing non-fruit and non-vegetables ingredients (e.g., pre-made salad containing chicken). We tagged all herbs and spices as "0."

Inclusion criteria	• "Plain" fresh fruits and vegetables and "plain" frozen fruits and vegetables.<br>• "Plain" canned fruits and vegetables items with ≤140 milligrams of sodium per serving.<br>• "Plain" prepared produce intended for off-premises consumption (e.g., pre-sliced mango).<br>• Canned and frozen fruits and vegetables with preservatives (e.g. citric acid) qualify if they also meet the above qualifications
Exclusion criteria	• Fruits and vegetables with added sugars (e.g., corn syrup), added fat (e.g., oil, cream, butter), and added non- fruit and vegetable food groups (e.g., grains, meat).

	◦ Use the ingredients list column to identify added sugar or added fat in canned and frozen fruits and vegetables using the following search terms: sugar, syrup, oil, cream, butter.<br>• Dried fruits and vegetables<br>• 100% fruit and vegetable juices, squeezables<br>• 100% fruit and vegetable “leather”<br>• Herbs and spices.
